# Pulmonary Marginal Zone B‐Cell Lymphoma of Mucosa‐Associated Lymphoid Tissue (PMZL‐MALT) Diagnosed by Radial Probe Endobronchial Ultrasound–Guided Biopsy: A Case Report

**DOI:** 10.1155/crpu/1058324

**Published:** 2026-05-22

**Authors:** Xia Ren, Wenjin Liang, Li Chen, Min Gao, Jing Wang, Nannan Pang, Chao Wu

**Affiliations:** ^1^ Department of Pulmonary and Critical Care Medicine, The First Affiliated Hospital of Shihezi University, Shihezi, China; ^2^ Department of Pathology, The First Affiliated Hospital of Shihezi University, Shihezi, China

**Keywords:** case report, pulmonary consolidation, pulmonary marginal zone B-cell lymphoma of mucosa-associated lymphoid tissue (PMZL-MALT), radial probe endobronchial ultrasound (RP-EBUS)

## Abstract

This case report presents a rare case of primary pulmonary marginal zone B‐cell lymphoma of mucosa‐associated lymphoid tissue (PMZL‐MALT). Follow‐up lung imaging performed 17 months after the initial presentation showed persistent pulmonary consolidation. After a radial probe endobronchial ultrasound (RP‐EBUS)–guided transbronchial lung biopsy, histopathological analysis confirmed the diagnosis of primary PMZL‐MALT. Notably, while RP‐EBUS is commonly used for solitary peripheral pulmonary lesions, its value in asymptomatic, bilateral, nonspecific consolidations—an easily misdiagnosed scenario often leading to unnecessary invasive procedures—remains underrecognized, which this case is aimed at highlighting. Our report is aimed at raising clinical awareness of this disease and promoting early diagnosis and treatment.

## 1. Introduction

Primary pulmonary lymphoma is a rare malignant tumor that arises from the lymphoid tissue within the lungs, without involvement of mediastinal or systemic lymph nodes, and constitutes only 0.5%–1% of primary pulmonary malignant tumors [1]. Pulmonary marginal zone B‐cell lymphoma of mucosa‐associated lymphoid tissue (PMZL‐MALT), clinically referred to as pulmonary MALT lymphoma, accounts for 90% of primary pulmonary lymphomas [2]. This disease is exceedingly rare. Due to the lack of specific clinical manifestations, imaging characteristics, and tumor markers, it is frequently misdiagnosed as pulmonary tuberculosis [3], pneumonia, lung cancer [4, 5], or other conditions in clinical practice.

Radial probe endobronchial ultrasound (RP‐EBUS) is a well‐established minimally invasive diagnostic tool for peripheral lung lesions; however, its clinical application is predominantly focused on symptomatic patients with solitary peripheral lung lesions or consolidations with a bronchial sign [6]. In contrast, the value of RP‐EBUS in asymptomatic patients with a long disease course and bilateral consolidations mimicking infection has not been sufficiently highlighted in clinical practice, making such cases particularly challenging to diagnose.

This case report focuses on an asymptomatic patient with a 17‐month disease course of bilateral pulmonary consolidations mimicking infection who was definitively diagnosed with PMZL‐MALT via RP‐EBUS–guided biopsy.

## 2. Case Presentation

**Figure 1:**
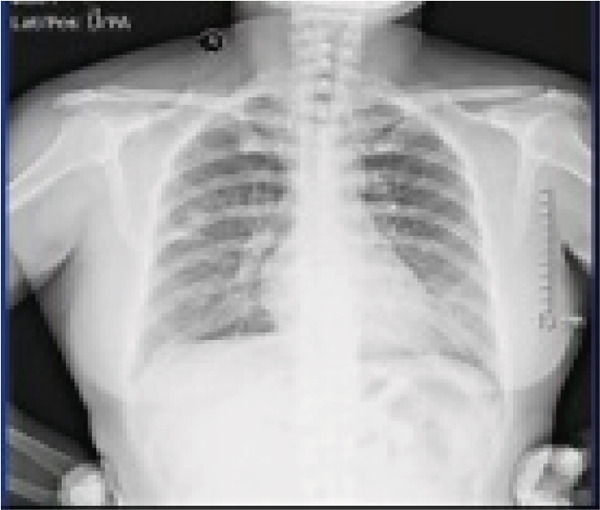
Anteroposterior chest scan in March 2024 shows increased density shadows in the middle field of the right lung and the lower field of the left lung.

**Figure 2:**
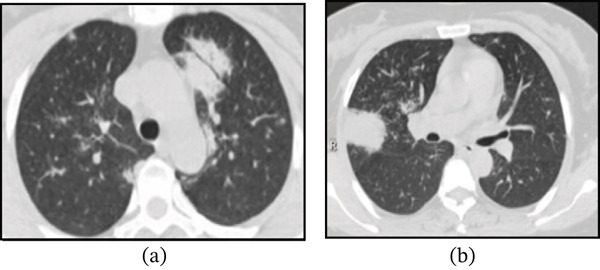
(a) Plain lung CT scan (August 2025) shows scattered patchy areas of increased density in both lungs; a prominent consolidation with visible air bronchograms is noted in the lingular segment of the left upper lobe. (b) Plain lung CT scan (August 2025) shows scattered patchy areas of increased density in both lungs; a significant consolidation is noted in the upper lobe of the right lung.

A 51‐year‐old female patient was admitted to our hospital with the chief complaint of “incidentally found abnormal lung imaging for 17 months, seeking further diagnosis.” The patient was completely asymptomatic throughout the entire disease course; the abnormal lung imaging was first detected incidentally during a routine health examination in March 2024 (Figure 1), not due to any respiratory (cough, sputum, hemoptysis, dyspnea, or chest pain) or systemic (fever, night sweats, or weight loss) symptoms. She sought medical attention solely to clarify the persistent abnormal imaging findings during the 17‐month follow‐up period (Figure 2a,b).

### 2.1. Smoking and Exposure History

The patient denied a history of active smoking and secondhand smoke exposure; denied a long‐term history of exposure to dust (such as asbestos or coal dust) and chemical substances (such as formaldehyde or benzene); and denied a history of raising poultry (such as parrots or pigeons) and close contact with tuberculosis patients.

Physical examination indicated symmetrical breathing movements in both lungs, symmetrical thoracic expansion, and symmetrical speech and fremitus. Coarse breath sounds were noted bilaterally, with no wet rales detected, and blood oxygen saturation was measured at 95%.

Relevant laboratory results were as follows: Inflammatory markers: routine blood test (white blood cell count 6.2 × 10^9^/L, neutrophil ratio 60.3%, lymphocyte ratio 32.1%), C‐reactive protein (CRP) 8.5 mg/L, procalcitonin (PCT) 0.08 ng/mL, and erythrocyte sedimentation rate (ESR) 15 mm/h were all within the normal range. Hepatitis B virus surface antigen: 4240 (elevated). Hepatitis B virus DNA: 1.501 × 10^5^ IU/mL (elevated). Lactate dehydrogenase (LDH): 185 U/L (normal). Alanine aminotransferase (ALT): 30 U/L (normal). Serum creatinine (Scr): 68.1 *μ*mol/L (normal). Tumor markers: carcinoembryonic antigen (CEA) 2.1 ng/mL (normal), squamous cell carcinoma antigen (SCC) 0.8 ng/mL (normal), neuron‐specific enolase (NSE) 12.3 ng/mL (normal), and cytokeratin 19 fragment assay 3.33 ng/mL (mildly elevated). The autoimmune workup included antinuclear antibody (ANA), extractable nuclear antigen (ENA) antibodies, rheumatoid factor (RF), and anticyclic citrullinated peptide, which were all within the normal range (Table 1).

**Table 1:** Table of investigations.

Examination category	Examination item	Result	Reference range
Inflammatory markers and routine blood test	White blood cell count	6.2 × 10^9^/L	4.0–10.0 × 10^9^/L
	Neutrophil ratio	60.3%	50.0%–70.0%
	Lymphocyte ratio	32.1%	20.0%–40.0%
	C‐reactive protein (CRP)	8.5 mg/L	< 10 mg/L
	Erythrocyte sedimentation rate (ESR)	15 mm/h	0–20 mm/h
	Procalcitonin (PCT)	0.08 ng/mL	0–0.15 ng/mL
Lactate dehydrogenase (LDH)	LDH	185 U/L	120–250 U/L
Hepatic and renal function	Alanine aminotransferase (ALT)	30 U/L	9–50 U/L
	Serum creatinine (Scr)	68.1 *μ*mol/L	44–110 *μ*mol/L
Tumor markers	Squamous cell carcinoma–associated antigen	2.1 ng/mL	0–2.7 ng/mL
	Carcinoembryonic antigen (CEA)	2.1 ng/mL	0–4.7 ng/mL
	Neuron‐specific enolase (NSE)	12.3 ng/mL	0–16.3 ng/mL
	Cytokeratin 19 fragment	3.33 ng/mL	0–3.3 ng/mL
	Hepatitis B virus surface antigen	4240	0–0.9
	Hepatitis B virus DNA	1.501 × 10^5^ IU/mL	< 1.0 × 10^2^ IU/mL
Autoimmune‐related examinations	Antinuclear antibody (ANA)	Negative	
	Extractable nuclear antigen (ENA) antibodies	Negative	
	Rheumatoid factor (RF)	Negative	
	Anticyclic citrullinated peptide antibody	Negative	

**Figure 3:**
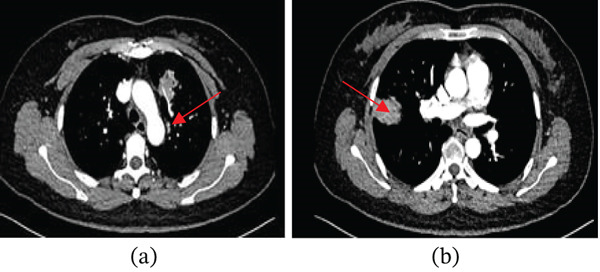
(a) Enhanced chest CT demonstrates significant enhancement of the lesion in the lingular segment of the left upper lobe, with visible vascular shadows within the lesion (arrow). (b) Enhanced chest CT demonstrates significant enhancement of the lesion in the right upper lobe.

Enhanced lung computed tomography (CT) indicated significant enhancement of the lesion in the tongue segment of the upper lobe of the left lung, with vascular shadows visible within the lesion. No enlarged lymph nodes were observed in the hilum or mediastinum of either lung (Figure 3a,b).

**Figure 4:**
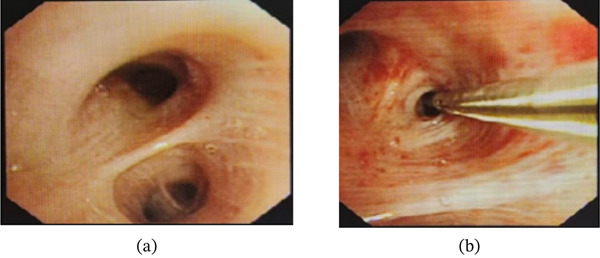
(a) Bronchoscopic image of the left upper lobe reveals no endobronchial neoplasms or mucosal abnormalities. (b) Radial probe endobronchial ultrasound (RP‐EBUS)–guided biopsy is performed on the lesion in the left upper lobe, achieving precise localization and successful tissue sampling for histopathological analysis.

**Figure 5:**
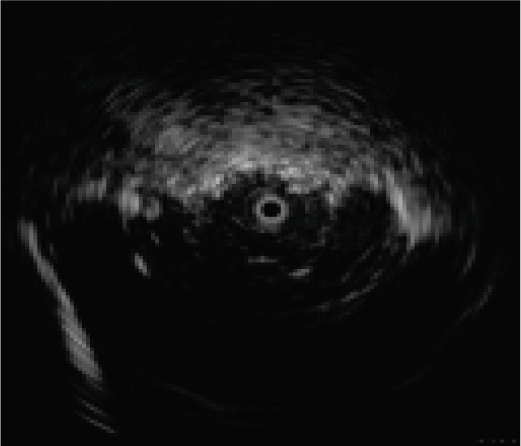
Radial probe endobronchial ultrasound (RP‐EBUS) image of the left upper lobe reveals abnormal echoes corresponding to the lesion.

**Figure 6:**
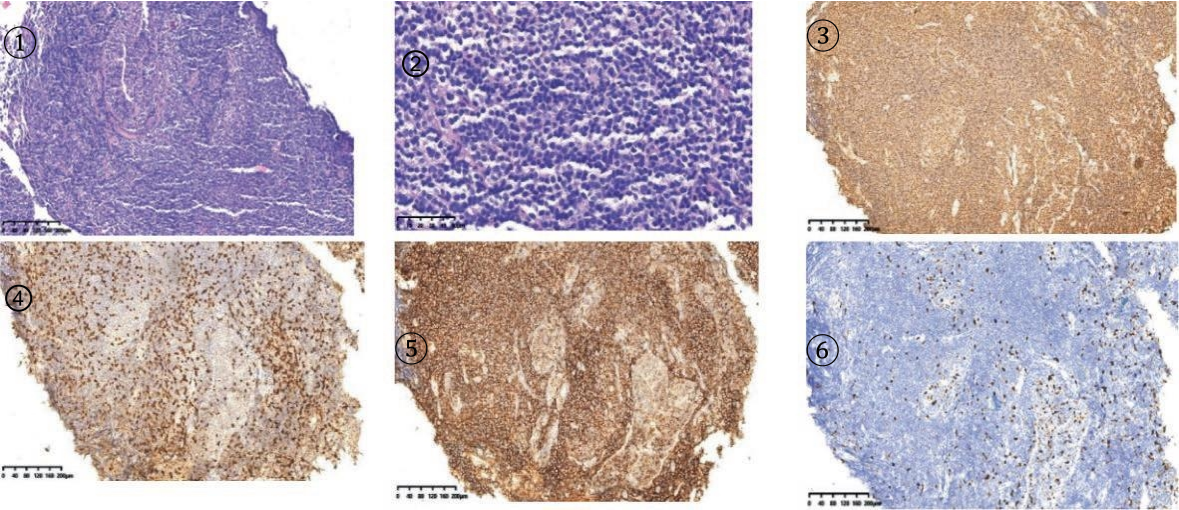
Pathological and immunohistochemical findings of the left upper lobe lesion. (1) Hematoxylin–eosin (HE) staining (×100). Numerous centrocyte‐like cells are observed. Tumor cells are small, with slightly irregular nuclei and little cytoplasm. (2) HE staining (×200). Mononuclear lymphocytes in the center of the mass exhibit diffuse growth, with cells of moderate size, disrupting the alveolar structure. (3) CD20 immunohistochemical staining (×100). Positive immunoreaction for CD20 is observed in the plasma membrane of tumor cells. (4) CD3 immunohistochemical staining (×100). Partial (focal) positive expression is observed, with scattered and focal positive signals predominantly located in the interstitial and peritumoral regions surrounding the lesion, with clear localization to the cytoplasm and cell membrane of T lymphocytes. No diffuse or strong positive expression is observed in neoplastic cells. (5) CD21 immunohistochemical staining (×100). Disrupted and fragmented follicular dendritic cell networks are observed within the lesion, with scattered, discontinuous positive signals distributed in the lymphoid infiltrate area. No intact lymphoid follicle structure is observed. (6) Ki‐67 immunohistochemical staining (×100). Ki‐67 expression in tumor cells is below 10%.

Electronic bronchoscopy revealed no neoplasms in the bronchi, and no mucosal abnormalities were noted (Figure 4a,b). Radial probe ultrasound bronchoscopy of the lingular branch in the upper lobe of the left lung displayed abnormal echoes (Figure 5). Pathological analysis of the RP‐EBUS–guided biopsy specimen revealed the following key histological features of PMZL‐MALT: (1) Dense lymphoid infiltration: dense and diffuse infiltration of small‐ to medium‐sized lymphoid cells was observed in the lung interstitium and around bronchial glands, without obvious nodular formation. (2) Small B‐cell phenotype: confirmed by immunohistochemical results, with CD20 (+), CD3 (partially +), CD5 (partially +), CD23 (focally +), Cyclin D1 (−), CD10 (−), BCL‐2 (+), BCL‐6 (weak +), MNDA (+), EBER (−), CD21 (+), and Ki‐67 (about 10% +) further confirming the diagnosis (HE staining, Figure 61,2; immunohistochemical staining, Figure 63–6). Bronchoalveolar lavage fluid was sent for mNGS (metagenomic next‐generation sequencing), tuberculosis smear, and routine bacterial culture, all of which were negative.

### 2.2. Staging, Treatment, and Follow‐Up

The patient underwent chest CT for disease staging but refused positron emission tomography–computed tomography (PET‐CT) due to personal and insurance reasons. Bone marrow aspiration and biopsy were not performed because the patient had normal LDH levels, had no clinical evidence of bone marrow infiltration, and refused further invasive examinations. After the definitive pathological diagnosis of pulmonary MALT lymphoma, the patient requested early discharge due to personal and insurance constraints, without receiving targeted treatment. We attempted to follow up with the patient through telephone and outpatient visits, but no effective follow‐up data have been obtained as of the revision of this manuscript.

## 3. Discussion

PMZL‐MALT is a rare, indolent extranodal non‐Hodgkin’s lymphoma with nonspecific clinical manifestations. The time span from the initial appearance of symptoms to confirmed diagnosis may be as long as several months to several years [7]. In this case, the patient had no clinical symptoms when abnormal lung imaging was first detected, which is consistent with the characteristics reported in previous literature [7]. Seventeen months later, a reexamination of chest CT showed multiple bilateral pulmonary consolidations, and the patient still had no respiratory symptoms such as cough, expectoration, chest tightness, chest pain, or hemoptysis at this time. This long‐term occult state of “asymptomatic” not only increases the difficulty of clinical vigilance but also poses great challenges to the differential diagnosis of PMZL‐MALT.

In terms of differential diagnosis, pulmonary infectious lesions must first be excluded. The patient had no clinical or laboratory evidence of acute infection, and mNGS, acid‐fast bacillus smear, and routine bacterial culture of bronchoalveolar lavage fluid were all negative, effectively ruling out infectious lesions. Combined with imaging features, chest CT of the patient showed multiple bilateral pulmonary consolidations and air bronchograms, and enhanced CT showed typical angiographic signs. Previous studies have shown that approximately 70% of PMZL‐MALT cases present with bilateral or multifocal lesions. Clinicians should be highly alert to the possibility of PMZL‐MALT when air bronchograms and vascular floating signs appear simultaneously on imaging [6].

For the patient with multiple bilateral pulmonary consolidations in this case, the conventional diagnostic path faced a dilemma: if CT‐guided percutaneous lung puncture was performed for the subpleural lesion in the right lung, although it is minimally invasive and has a relatively high diagnostic rate, the patient still had a risk of pneumothorax and postoperative hemoptysis; if targeting the left lung consolidation, the limitations of conventional bronchoscopy needed to be considered. Minezawa et al. [8] found that air bronchograms on CT are a predictive indicator that significantly improves the positive rate of bronchoscopic diagnosis. The overall diagnostic rate of RP‐EBUS for peripheral pulmonary lesions is 70% [9]. After comprehensive consideration, our team performed left lung RP‐EBUS for the patient, achieved precise localization under real‐time ultrasound guidance, and obtained sufficient tissue samples. RP‐EBUS–guided biopsy yielded sufficient tissue samples to identify the essential histological features of PMZL‐MALT, including dense lymphoid infiltrate, lymphoepithelial lesions, and a small B‐cell phenotype, along with a typical immunophenotype (CD20+, CD3 partially +, CD5 partially +, CD10−), which laid the foundation for a definitive diagnosis, thereby avoiding the need for the patient to undergo percutaneous lung puncture biopsy. After a confirmed diagnosis, the patient was discharged voluntarily due to medical insurance issues, and the later development direction could not be monitored.

It is worth noting that the patient had a history of chronic hepatitis B. Although studies have shown a clear association between chronic hepatitis C virus (HCV) infection and marginal zone lymphoma (MZL) [10], through a systematic literature review, there is currently no conclusive evidence proving a direct causal relationship between chronic hepatitis B and the occurrence of PMZL‐MALT. The potential association between the two is worthy of further large‐sample, multicenter studies in the future.

In conclusion, PMZL‐MALT patients have nonspecific clinical manifestations. For patients with long‐term pulmonary consolidations, especially those with air bronchograms and angiographic signs on imaging, timely performance of RP‐EBUS–guided lung biopsy, combined with histopathological, immunohistochemical staining and genetic testing analysis, is an effective method to reduce the misdiagnosis rate.

## Author Contributions

Xia Ren designed the study and wrote the manuscript. Wenjin Liang, Li Chen, Min Gao, Jing Wang, Nannan Pang, and Chao Wu collected clinical data, created the figures, and revised the manuscript.

## Funding

This work was supported by the Key R&D Project of the Corps (2024AB066).

## Disclosure

All authors read and approved the final manuscript.

## Ethics Statement

The authors have nothing to report.

## Consent

All efforts have been made to avoid including any patient identifiers in this case report. Patient consent could not be obtained, as multiple attempts to contact the patient were unsuccessful.

## Conflicts of Interest

The authors declare no conflicts of interest.

## Data Availability

No datasets were generated or analyzed during the current study.
